# Supplementary material for: High Expression of NT5DC2 Is a Negative Prognostic Marker in Pulmonary Adenocarcinoma
Source: Cancers (Basel). 2022 Mar 9;14(6):1395. doi: 10.3390/cancers14061395 (PMC8946072; doi:10.3390/cancers14061395)
Supplement: Supplementary file 1 [file cancers-14-01395-s001.zip › Figure S1.pdf]

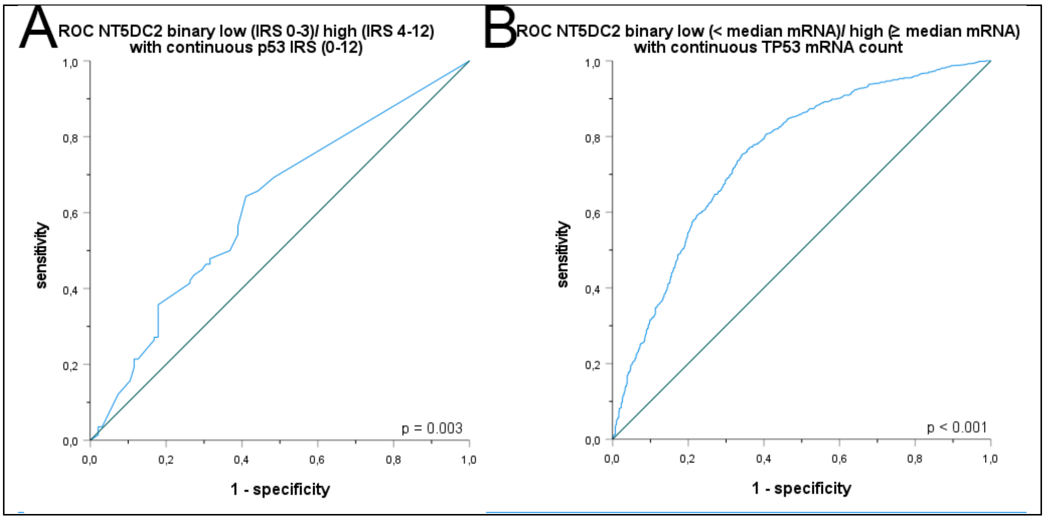

Figure S1. Receiver-operating characteristic analysis of binary NT5DC2 expression with continuous IRS (0-12) of p53 in the protein expression analysis cohort (**A**) and binary NT5DC2 expression with continuous mRNA TP53 count in the gene expression analysis cohort (**B**).
