# Supplementary material for: High Expression of NT5DC2 Is a Negative Prognostic Marker in Pulmonary Adenocarcinoma
Source: Cancers (Basel). 2022 Mar 9;14(6):1395. doi: 10.3390/cancers14061395 (PMC8946072; doi:10.3390/cancers14061395)
Supplement: Supplementary file 1 [file cancers-14-01395-s001.zip › Figure S2.pdf]

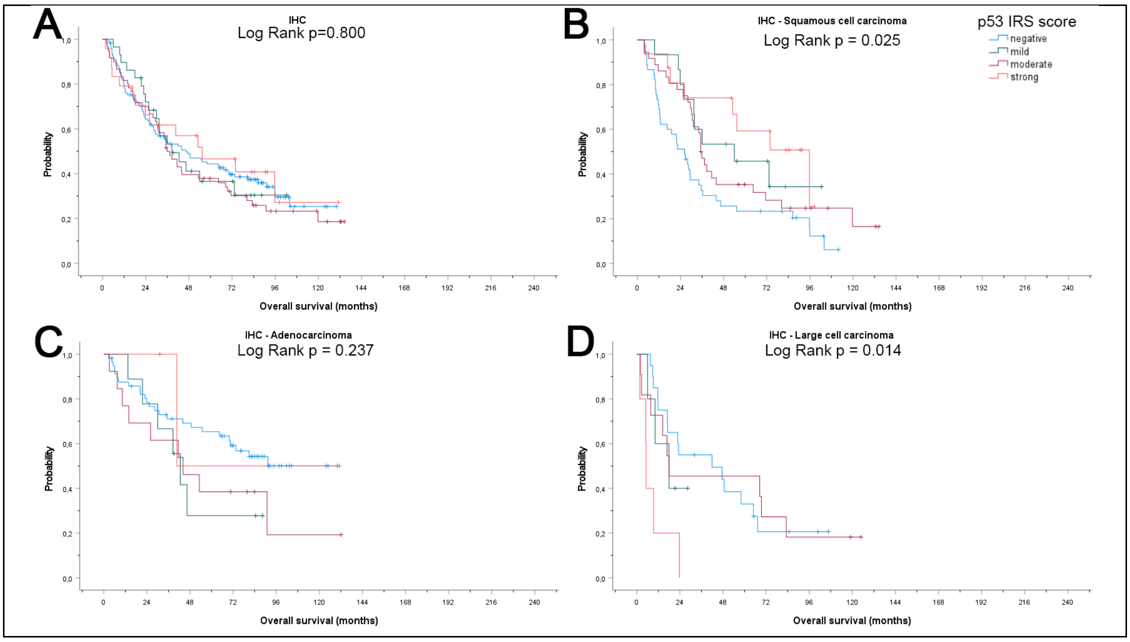

Figure S1. Kaplan-Meier curves of univariate survival regarding nuclear p53 IHC stain. IRS score is split by negative (IRS 0-1), mild (IRS 2-3), moderate (IRS 4-8) and strong (IRS 9-12). Analyses present survival in the overall cohort (**A**), squamous cell carcinoma (**B**), adenocarcinoma (**C**) and large cell carcinoma (**D**). In contrast to adenocarcinoma, in squamous cell carcinoma an overexpression of nuclear p53 results in beneficial survival.
