# Supplementary material for: High Expression of NT5DC2 Is a Negative Prognostic Marker in Pulmonary Adenocarcinoma
Source: Cancers (Basel). 2022 Mar 9;14(6):1395. doi: 10.3390/cancers14061395 (PMC8946072; doi:10.3390/cancers14061395)
Supplement: Supplementary file 1 [file cancers-14-01395-s001.zip › Figure S3.pdf]

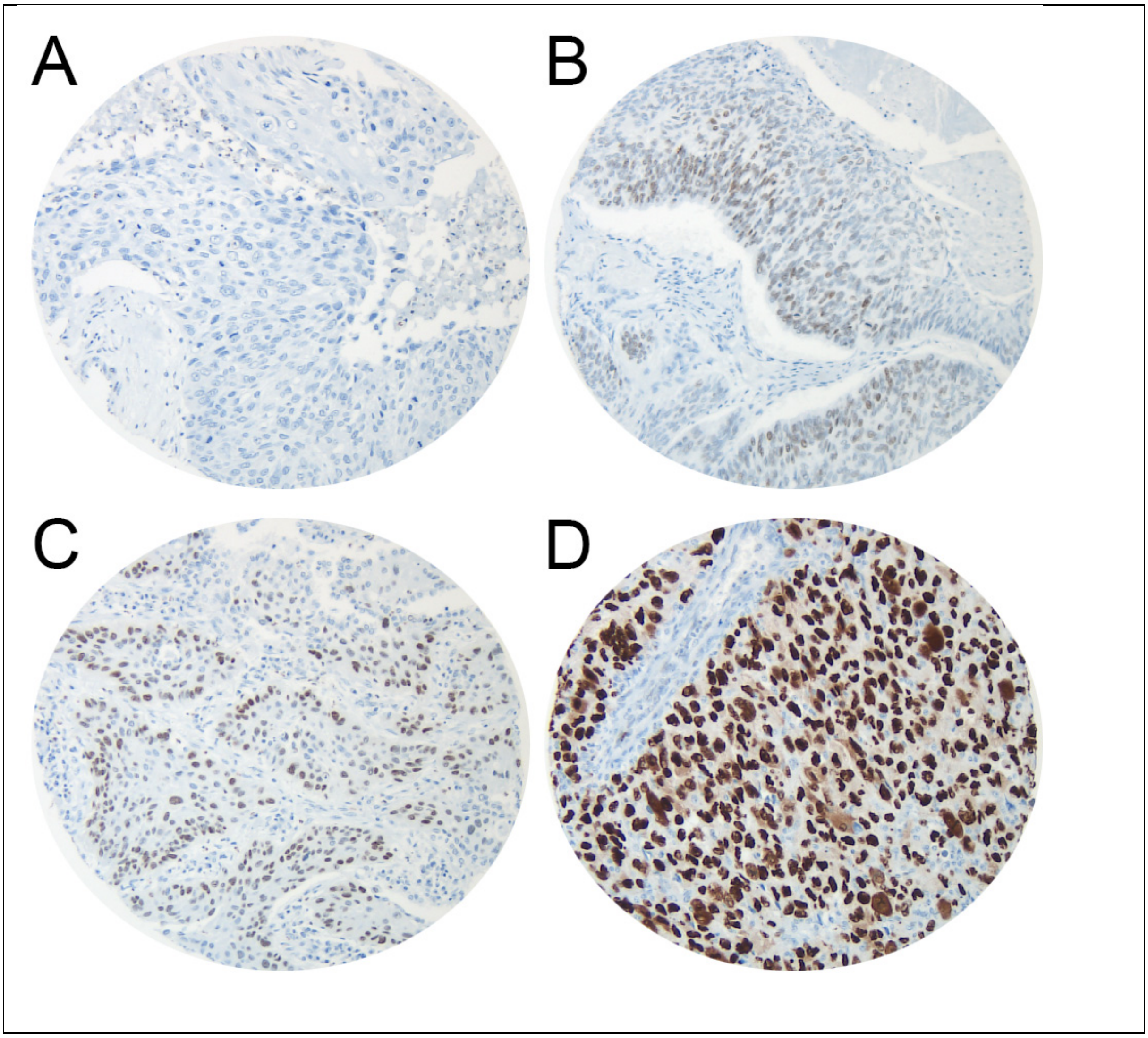

Figure S1. Immunohistochemistry of p53 in NSCLC TMAs. Primary antibody was Roche/ Ventana 790-2912 mouse monoclonal IgG_1 anti-p53 (DO-7) antibody. Secondary antibody was Roche/Ventana 760-700 OptiView DAB IHC Detection Kit. Analyses were performed on an Olympus BX51 microscope with an average magnification of x200. (**A**) represents a negative core (IRS 0), (**B**) reveals an IRS intensity 1 core (IRS 1), (**C**) is an intensity 2 core (IRS 2) and (**D**) is an intensity 3 core (IRS 3), respectively.
