# Supplementary material for: High Expression of NT5DC2 Is a Negative Prognostic Marker in Pulmonary Adenocarcinoma
Source: Cancers (Basel). 2022 Mar 9;14(6):1395. doi: 10.3390/cancers14061395 (PMC8946072; doi:10.3390/cancers14061395)
Supplement: Supplementary file 1 [file cancers-14-01395-s001.zip › Table S1.pdf]

Table S1. Immunohistochemical IRS-Score of NT5DC2 and p53.

	NT5DC2 Expression								
	**Negative IRS 0-1**		**Mild IRS 2-3**		**Moderate IRS 4-8**		**Strong IRS 9-12**		***p*-Value**
	**n**	**in %**	**n**	**in %**	**n**	**in %**	**n**	**in %**	
**p53 Expr.**	36		59		123		17		0.016
Negative IRS 0-1	26	72.2	32	54.2	54	43.9	10	58.8	
Mild IRS 2-3	4	11.1	8	13.6	16	13.0	1	5.9	
Moderate IRS 4-8	5	13.9	13	22.0	38	30.9	4	23.5	
Strong IRS 9-12	1	2.8	6	10.2	15	12.2	2	11.8	

p-value: Kruskal-Wallis test.
