# Supplementary material for: High Expression of NT5DC2 Is a Negative Prognostic Marker in Pulmonary Adenocarcinoma
Source: Cancers (Basel). 2022 Mar 9;14(6):1395. doi: 10.3390/cancers14061395 (PMC8946072; doi:10.3390/cancers14061395)
Supplement: Supplementary file 1 [file cancers-14-01395-s001.zip › Table S2.pdf]

Table S2. Association of NT5DC2 protein expression with tumor grade.

Protein Expression Cohort	NT5DC2 Protein Expression	Low Grade (G1/2)		High Grade (G3/4)		*p*-Value *
		n	in %	n	in %	
Overall cohort	Low (IRS 0-3)	42	53.2 %	65	38.2 %	$p = 0.029$
	High (IRS 4-12)	37	46.8 %	105	61.8 %	
SCC	Low (IRS 0-3)	22	68.8 %	33	37.1 %	$p = 0.003$
	High (IRS 4-12)	10	31.3 %	56	62.9 %	
ADC	Low (IRS 0-3)	19	42.2 %	18	40.9 %	$p = 1.000$
	High (IRS 4-12)	26	57.8 %	26	59.1 %	

SCC: squamous cell carcinoma, ADC: adenocarcinoma, * *p*-values deriving the Fisher's exact test.
